# High Fat Diet and *In Utero* Exposure to Maternal Obesity Disrupts Circadian Rhythm and Leads to Metabolic Programming of Liver in Rat Offspring

**DOI:** 10.1371/journal.pone.0084209

**Published:** 2014-01-09

**Authors:** Sarah J. Borengasser, Ping Kang, Jennifer Faske, Horacio Gomez-Acevedo, Michael L. Blackburn, Thomas M. Badger, Kartik Shankar

**Affiliations:** 1 Arkansas Children's Nutrition Center, Little Rock, Arkansas, United States of America; 2 Department of Pediatrics, University of Arkansas for Medical Sciences, Little Rock, Arkansas, United States of America; Institute of Medical Research A Lanari-IDIM, University of Buenos Aires-National Council of Scientific and Technological Research (CONICET), Argentina

## Abstract

The risk of obesity in adulthood is subject to programming beginning at conception. In animal models, exposure to maternal obesity and high fat diets influences the risk of obesity in the offspring. Among other long-term changes, offspring from obese rats develop hyperinsulinemia, hepatic steatosis, and lipogenic gene expression in the liver at weaning. However, the precise underlying mechanisms leading to metabolic dysregulation in the offspring remains unclear. Using a rat model of overfeeding-induced obesity, we previously demonstrated that exposure to maternal obesity from pre-conception to birth, is sufficient to program increased obesity risk in the offspring. Offspring of obese rat dams gain greater body weight and fat mass when fed high fat diet (HFD) as compared to lean dam. Since, disruptions of diurnal circadian rhythm are known to detrimentally impact metabolically active tissues such as liver, we examined the hypothesis that maternal obesity leads to perturbations of core clock components and thus energy metabolism in offspring liver. Offspring from lean and obese dams were examined at post-natal day 35, following a short (2 wk) HFD challenge. Hepatic mRNA expression of circadian (CLOCK, BMAL1, REV-ERBα, CRY, PER) and metabolic (PPARα, SIRT1) genes were strongly suppressed in offspring exposed to both maternal obesity and HFD. Using a mathematical model, we identified two distinct biological mechanisms that modulate PPARα mRNA expression: i) decreased mRNA synthesis rates; and ii) increased non-specific mRNA degradation rate. Moreover, our findings demonstrate that changes in PPARα transcription were associated with epigenomic alterations in H3K4me3 and H3K27me3 histone marks near the PPARα transcription start site. Our findings indicated that offspring from obese rat dams have detrimental alternations to circadian machinery that may contribute to impaired liver metabolism in response to HFD, specifically via reduced PPARα expression prior to obesity development.

## Introduction

The epidemic rise in obesity incidence across the age spectrum continues to be a daunting public health challenge. Most concerning is the prevalence of obesity in infants and children as obesity in childhood tracks strongly into adulthood [1]. It is estimated that infants in the United States (birth to 2 y) are twice as likely to be greater than the 95^th^ percentile for weight-to-length measurements, indicating an increasing number of infants weigh more than is considered healthy [2]. Maternal obesity and excessive gestational weight gain have been identified as factors that contribute to increased obesity in the offspring. This is particularly significant, as over 60% of all pregnancies in the United States are in women who are either overweight or obese at conception [3]. Several studies have shown strong associations between maternal OB and obesity in the offspring, both in childhood [4]–[7] and in adulthood [8]–[10]. A recent study of 37,000 individuals showed greater risk of cardiovascular disease and pre-mature death in those born to OB women [11]. Likewise, the diminished risk of obesity in children born to OB women who lost weight prior to pregnancy, also strongly suggests a role for developmental programming [12], [13]. In animal models, exposure to maternal obesity unambiguously influences the risk of obesity in the offspring [14]–[18]. Collectively, the evidence suggests that susceptibility to obesity may begin prior to birth. Consequently, understanding how the intrauterine environment contributes to offspring obesity development is a pertinent health concern.

The Barker hypothesis offers a framework linking the *in utero* environment to long-term health outcomes in offspring [19]. While originally described in low-birth weight infants, it is now clear that maternal caloric excess and high fat diets also engender similar long-term programming of offspring obesity risk [20]. Both epidemiological [12], [13], [21] and animal [22]–[25] studies demonstrate intrauterine exposure to maternal obesity is associated with increased adiposity and weight gain. We, as well as others, have reported metabolic programming due to maternal nutrition which includes insulin resistance [7], [22], cardiovascular disease [26], [27], nonalcoholic fatty liver disease (NAFLD) [28], [29], decreased energy expenditure [30], and impaired ability to utilize fatty acids for energy in offspring [30]. Notably, our model limits the exposure of maternal obesity exclusively to the intrauterine environment through cross-fostering of offspring to lean surrogate dams at post-natal day (PND) 1 [22]. Our studies have also shown that impaired energy metabolism in obese dam offspring occurs prior to alterations in body weight and adiposity [28], [30]. Although many such advances have furthered our understanding of fetal programming, much of the molecular mechanisms leading to metabolic dysregulation remain to be elucidated.

A close relationship exists between metabolism and diurnal circadian rhythm, in which disruption promotes obesity and metabolic disease [31]–[35]. Perturbations in circadian rhythms are known to impact energetics, particularly in tissues regulating metabolism (considered peripheral clocks) such as liver, skeletal muscle, brown adipose, and white adipose [36]–[38]. Factors such as feeding, fasting, or type of diet can modulate rhythmicity and metabolism. More specifically, recent studies suggest that circadian rhythms are affected by gestational exposure to dietary manipulations such as protein restriction [39] or high fat consumption [40]. We have previously demonstrated that exposure to maternal obesity disrupted targets downstream of peroxisome proliferator-activated receptor (PPAR)α and AMPK which are key regulators responsible for orchestrating fatty acid oxidation prior to obesity development [28]. Since, PPARα expression oscillates in a circadian fashion throughout the day, and also acts as a circadian regulator [41]–[43], it remains unclear whether maternal obesity impairs PPARα signaling via disruption of circadian rhythm.

In the present study, we examined the hypothesis that intrauterine exposure to maternal obesity disrupts offspring circadian rhythm and liver metabolism following a short term high fat diet (HFD) challenge after weaning. First, we investigated mRNA expression of core clock components which included circadian locomotor output cycles kaput (CLOCK), brain and muscle ARNTL-like protein-1 (BMAL1), REV-ERBα, Cryptochromes (CRYs), and Periods (PERs). Second, we applied a mathematical model to identify distinct mechanisms that contribute to changes in PPARα mRNA expression due to maternal obesity and HFD. Lastly, we investigated histone modifications in the PPARα promoter to elucidate epigenetic mechanisms regulating PPARα expression. Our results demonstrate that offspring from obese rat dams have detrimental alterations to circadian machinery that contribute to impaired liver metabolism in response to high fat feeding.

## Materials and Methods

### Animals and chemicals

Female Sprague-Dawley rats (150–175 g) were obtained from Charles River Laboratories (Wilmington, MA). Animals were housed in an AAALAC-approved animal facility in a temperature and light controlled room (12 h light-12 h dark cycle). All experimental protocols were approved by the Institutional Animal Care and Use Committee at the University of Arkansas for Medical Sciences (Protocol # 2971). Unless specified, all chemicals were obtained from Sigma-Aldrich Chemical Co. (St. Louis, MO).

### Experimental protocol

**Figure 1:**
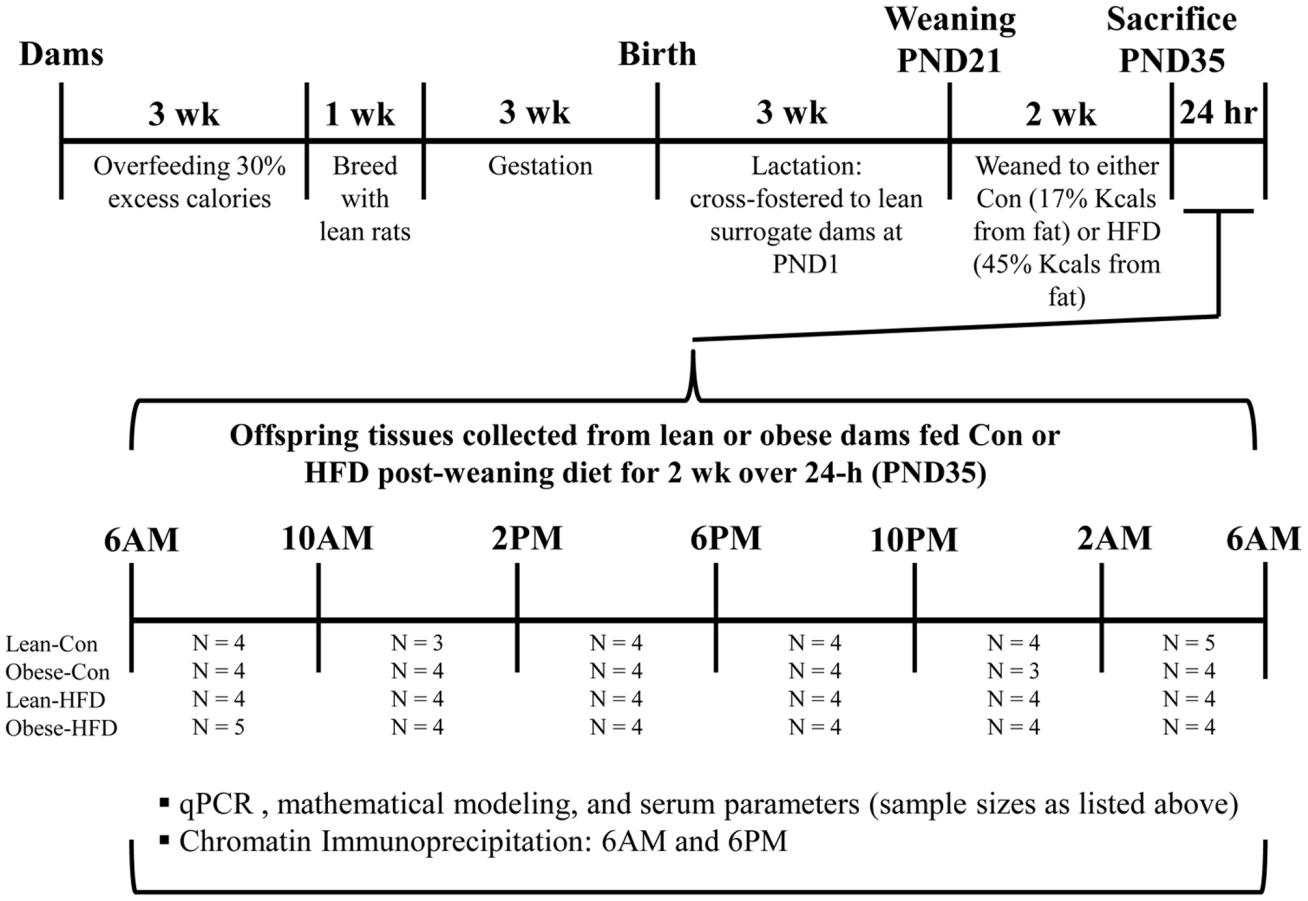
Schematic of experimental design, interventions and outcomes.

Virgin female Sprague-Dawley rats were intragastrically cannulated to receive total enteral nutrition (TEN) at age 8 wk and allowed to recover for 10 d as previously described [22], [44]–[47]. Rats were fed liquid TEN diets at either 155 kcal/kg^3/4^·d (referred to as **lean dams**) or at 220 kcal/kg^3/4^·d (40% excess calories, referred to as **obese dams**). We have previously reported body weights and body compositions of lean and obese dams [22]. TEN diets met National Research Council (NRC) nutrient recommendations as used previously by our group [22], [44], [46]–[50] and consisted of 20% protein (casein), 75% carbohydrate (dextrose and maltodextrin), and 5% fat (corn oil) . Infusion of diets was carried out for 3 wk allowing for precise control of both diet composition and caloric intake in a low-stress manner. Animals had *ad libitum* access to drinking water and body weights were measured three times per week. Following 3 wk of overfeeding to induce obesity in the 220 kcal/kg^3/4^·d group, lean and obese rats (N = 15/group) were allowed to mate for 1 wk. Each female rat was housed with one lean breeder male and allowed *ad libitum* access to AIN-93G diet during this period. After mating all female rats (lean and obese) received diets at 220 kcal/kg^3/4^·d (NRC recommended caloric intake for pregnancy in rats). All rats were allowed to give birth naturally. Numbers and sex of pups, birth weight, and crown-to-rump and anogenital distance were measured for each pup on PND1 as previously described [22], [28]. On PND1, four male and four female pups from each litter were cross-fostered to lean dams that had been previously time-impregnated to give birth on the same day as the obese dams receiving infusion diets. Cross-fostered dams were not cannulated and had *ad libitum* access to AIN-93G pelleted diets throughout lactation. Using this experimental paradigm, we ensured that offspring's exposure to any effects of maternal obesity was limited exclusively to the intrauterine environment [22]. Female offspring of lean and obese dams were used for separate experiments, and only data from male offspring are reported here. At PND21 male offspring were weaned onto either an AIN-93G (17% kcals fat) or high fat diet (45% kcals fat) for 2 wk. Offspring were weighed and sedated with carbon dioxide and euthanized via exsanguination in the fed condition at PND35 every 4 h over a 24 h period (N = 4/group for all time points except, N = 5/group for Lean-Con 2AM and Obese-HFD 6AM and N = 3/group for Lean-Con 10PM and Obese-Con 10PM, See **Figure 1** for detailed offspring grouping). Each offspring group represents 2–4 distinct biological litters. At sacrifice, liver was weighed and immediately frozen in liquid nitrogen and stored at −70°C for later analyses. Serum was obtained by centrifugation of blood samples and stored at −20°C. Animal characteristics and serum parameters are shown in **Table S2 in File S1 and Figure S1 in File S1**.

### Real-time RT-PCR

Total RNA was isolated from liver of offspring at PND35 (N = 3–5 rats per group per time point) using RNeasy mini columns (QIAGEN, Valencia, CA) including on-column DNase digestion. One microgram of total RNA was reverse transcribed using iScript cDNA synthesis kit (BioRad, Hercules, CA). Real-time PCR analysis was performed as described previously using an ABI Prism 7500 Fast instrument (Carlsbad, CA) [50], [51]. Area under the curve (AUC) was calculated using the trapezoidal rule. Gene specific primers were designed using Primer Express Software (**Table S1 in File S1**). Relative amounts of mRNA were quantified using a standard curve and normalized to the expression of SRP14 mRNA.

### Mathematical Modeling

The mammalian circadian cycle has been computationally modeled by Leloup and Golbeter under normal physiological conditions [52], [53]. Here, we extended their model to analyze the effect of maternal obesity and HFD on PPARα mRNA expression. The relative mRNA expression level of PPARα was analyzed with a differential equation that has the form:
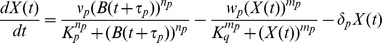
where *X(t)* represented the relative gene expression level of PPARα. A more detailed description of the model is described in **Table S3 in File S1**. Parameter values were obtained by Monte Carlo simulations (n = 10,000) followed by a conjugate-gradient method implemented in Mathematica 8 (Wolfram Research Inc, Champaign, IL) as shown in **Table S4 in File S1**. To assess the reliability and robustness of the model, we performed a sensitivity analysis using Partial Rank Correlation Coefficient (PRCC) method at two switching points of the dark/light cycle that are critical for the circadian cycle, namely 6AM and 6PM with sample size set to N = 1000 [54].

### Chromatin Immunoprecipitation (ChIP)

Histone modifications on the PPARα promoter were assessed using ChIP. Samples were processed according to a previously established protocol [55] and using a ChIP-IT enzymatic kit (Active Motif, Carlsbad, CA) with minor modifications as described previously [56]. Briefly, pools of liver samples (each pool representing 4–5 separate animals) at 6AM and 6PM, from offspring of lean or obese dams fed either control or HFD, were used for analyses. Samples were minced and fixed in a 1% formaldehyde solution and dounce homogenized. Nuclear isolation was performed and chromatin was sheared using a Covaris S220 focused-ultrasonicator (Woburn, MA). MagnaChIP protein A/G beads (Millipore, Billerica, MA) and chromatin were pre-blocked with 0.5% BSA and used to pre-clear the chromatin. Immunoprecipitation was performed using 2.5 µg of ChIP grade antibodies for H3K4me3 (Active Motif, Carlsbad, CA), H3K27me3 (Millipore, Billerica, MA), or matched nonspecific IgG (Millipore, Billerica, MA). Target binding regions on PPARα promoter were amplified via qPCR using an Eco Real-Time PCR System (Illumina, San Diego, CA), ±500 base pairs from the transcription start site (TSS) (**See Table S1 in File S1 for sequences**).

### Statistical Analysis

Data are expressed as means ± SEM, significance was set at *p*<0.05. Differences in mRNA expression between offspring of lean and obese dams at each time point at PND35 were determined using two-tailed Student's *t*-test. Differences between offspring of lean and obese dams at PND35 fed AIN-93G or HFD for all other measured parameters were analyzed using two-way analysis of variance (ANOVA). Significant interactions identified by two-way ANOVA were followed by a one-way ANOVA and all pair-wise comparisons by Student-Newman-Keuls. Statistical analyses were performed using SigmaPlot 12.5 software (Systat Software Inc., San Jose, CA).

## Results

### Maternal obesity and high fat diet disrupt hepatic mRNA amplitude of clock machinery in offspring

**Figure 2:**
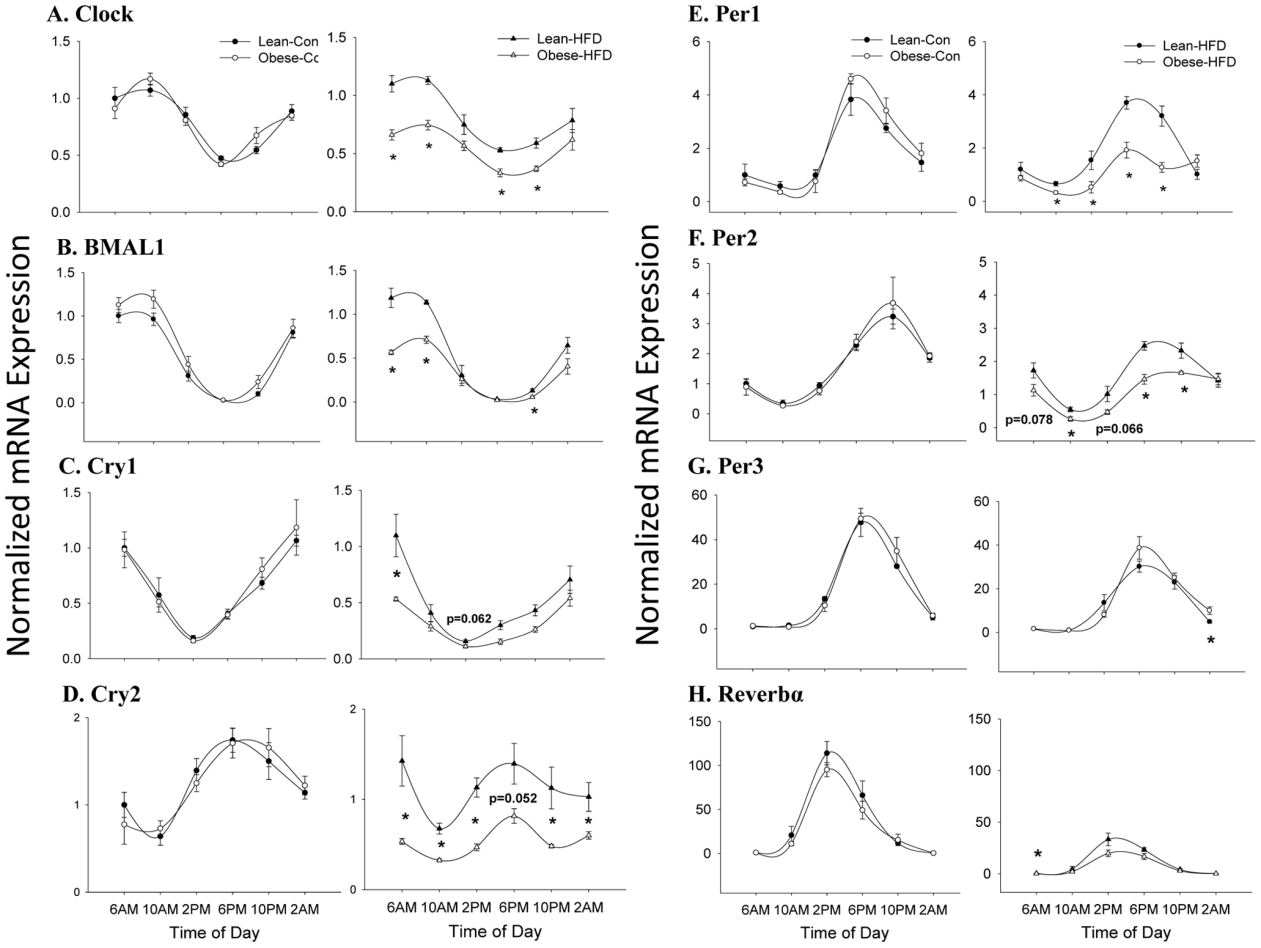
Hepatic mRNA expression of core circadian genes. mRNA expression was assessed in lean and obese dam offspring (A–H) fed either control or high fat diets (HFD) from weaning through PND35 (N = 3–5 animals per group, Lean-Con 2AM and Obese-HFD 6AM (N = 5 per group), Lean-Con 10AM and Obese-Con 10PM (N = 3 per group), and all the remaining groups (N = 4)). Gene expression was assessed via real-time RT-PCR. All genes were normalized to SRP14 and expressed relative to Lean-Con at 6AM. Data are presented as mean ± SEM Statistical differences were determined using a Student's *t* test. * denotes significance, P<0.05.

We examined mRNA expression of the core clock components (CLOCK, BMAL1, REV-ERBα, Per1, Per2, Per3, Cry1, and Cry2) to evaluate if maternal obesity and/or exposure to HFD altered circadian rhythm over a 24 h period (**Figure 2**). The clock machinery forms a transcriptional-translational loop (TTL) where BMAL1 dimerizes with CLOCK forming a heterodimer that binds to E-box promoter elements to positively regulate CLOCK and clock controlled genes [32]. As expected, mRNA expression of CLOCK and BMAL1 resembled each other in their cyclic oscillations in which both peaked at 10AM and steadily declined until 6PM, when the lowest levels of expression occurred as depicted in **Figure 2A and 2B**. Maternal obesity did not alter rhythmicity or levels of mRNA expression of either CLOCK or BMAL1 in control-diet fed offspring of lean and obese dams. However, in HFD-fed offspring AUC for CLOCK and BMAL1 mRNA were lowest in offspring from obese dams indicated by a significant interaction of maternal obesity and HFD consumption (p<0.001) (**Table 1**).

**Table 1:** 24(AUC) (N = 3–5/group).

Target Gene Normalized to SRP14					*P* Values		
	Offspring of Lean Dams		Offspring of Obese Dams		Maternal Obesity x Postweaning Diet	Effect of Maternal Obesity	Effect of Postweaning HFD
	Control	HFD	Control	HFD			
Core Clock Machinery							
CLOCK	23.7±0.6^a^	24.0±0.8^a^	23.7±0.6^a^	16.0±0.3^b^	**<0.001**	**0.002**	**<0.001**
BMAL1	15.2±0.9^a^	15.7±0.8^a^	17.8±0.8^a^	9.4±0.3^b^	**<0.001**	0.686	**0.001**
Reverbα	1188±116	396±27	1043±83	251±37	0.885	**0.050**	**<0.001**
Per1	53.7±1.9^a^	61.5±2.8^a^	62.6±5.9^a^	30.4±1.7^b^	**<0.001**	0.239	**0.012**
Per2	47.0±1.4^a^	47.9±3.6^a^	48.7±3.7^a^	30.4±1.4^b^	**0.019**	0.150	**0.036**
Per3	550±59	422±23	585±35	450±45	0.982	0.489	**0.027**
Cry1	18.1±1.3^a^	13.6±0.6^b^	17.3±1.2^a^	8.1±0.4^c^	**0.040**	**0.036**	**<0.001**
Cry2	37.3±1.7^a^	33.7±1.4^a^	35.9±1.3^a^	15.9±0.7^b^	**<0.001**	**<0.001**	**<0.001**
Metabolic and Epigenetic Regulators							
PPARα	60.5±2.4^a^	24.6±2.2^b^	59.7±2.3^a^	12.1±0.7^c^	**0.024**	**<0.049**	**<0.001**
EZH2	26.9±2.1^a^	29.4±6.3^a^	27.6±2.0^a^	56.0±6.5^b^	**0.001**	**0.002**	**0.001**
SIRT1	30.3±1.2^a^	25.7±0.6^b^	31.5±1.2^a^	17.2±0.6^c^	**<0.001**	**0.047**	**<0.001**

Area under the curve was assessed using the trapezoidal rule. Statistical differences were determined using a two-way ANOVA examining the effects of maternal obesity and post-weaning HFD. Significant interactions identified by two-way ANOVA were followed by a one-way ANOVA and all pair-wise comparisons by Student-Newman-Keuls. Data are expressed as mean ± SEM, **bold** values represent significant main effects and interactions. Values with different letters (^a,b,c^) are significantly different from each other (P<0.05).

Similar to CLOCK/BMAL1, Cry and Per also form a heterodimer, which acts as a negative regulator by impairing the action of CLOCK/BMAL1 to complete the TTL. As anticipated, Cry2 and Per 1, 2, and 3 all exhibited mRNA expression patterns that were antiphasic to that of CLOCK/BMAL1 as these targets peaked at 6PM and reached their lowest levels at 10AM (**Figure 2D–G**). Per2 mRNA expression of control-fed lean and obese dam offspring displayed slightly shifted expression, peaking occurred at 10PM as opposed to 6PM (**Figure 2F**). Similar to CLOCK/BMAL1, maternal obesity by itself did not alter mRNA expression of Cry (1, 2) and Per (1,2,3), but only in combination with HFD did maternal obesity show an effect of further reducing expression levels of Cry and Per (**Table 1**). Cry1 did not follow an anti-phasic pattern of mRNA expression to CLOCK/BMAL1, instead Cry1 was highest at 6AM and 2AM and was lowest at 2PM as shown in **Figure 2C**.

Two nuclear receptors are central to the regulation of the above mentioned clock components via control of BMAL1 transcription. Retinoic acid receptor-related orphan receptor (ROR)α is thought to activate BMAL1 transcription, while REV-ERBα suppresses it. In the present study, we found that RORα mRNA expression did not differ between groups (data not shown). However, REV-ERBα mRNA expression was robustly induced at 2 PM in lean-CON (∼114-fold) and obese-CON (∼95-fold) relative to 6AM (**Figure 2H**). However, there effects were severely blunted in high fat-fed offspring via a decrease of ∼81-fold in lean dam offspring and ∼75-fold in obese dam offspring in mRNA expression amplitude as compared to control-fed counterparts (**Figure 2H**). This dramatic HFD-induced reduction of REV-ERBα mRNA expression did not coincide with substantial changes in BMAL1 transcription in HFD-fed offspring suggesting BMAL1 transcription may be regulated via other mechanisms.

### Disruption of circadian machinery alters PPARα in obese dam offspring

**Figure 3:**
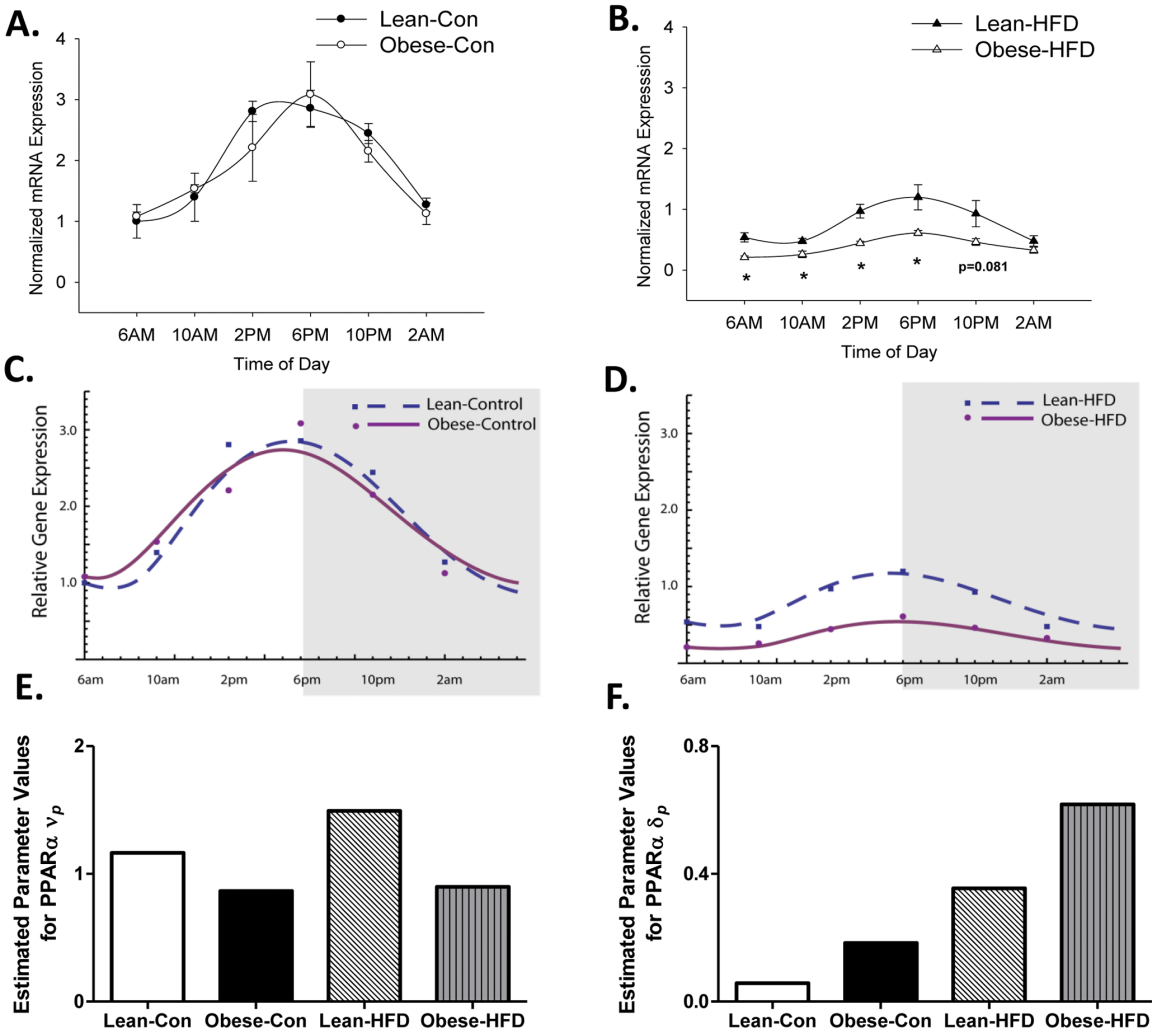
Circadian expression of PPAR-α mRNA. Hepatic PPARα mRNA was assessed in lean and obese dam offspring fed A) control or B) HFD at PND35 (N = 3–5 animals per group, Lean-Con 2AM and Obese-HFD 6AM (N = 5 per group), Lean-Con 10AM and Obese-Con 10PM (N = 3 per group), and all the remaining groups (N = 4)). Statistical differences were determined using a Student's *t* test. * denotes significance, P<0.05. Mathematical model derived from relative PPARα mRNA expression of offspring of lean and obese dams fed C) control diet or D) HFD at PND35. Model fitting incorporated eight parameters representing distinct biological mechanisms. E) Optimal values for the parameter *ν_p_* which represents rates of PPARα mRNA synthesis and F) *δ_p_* which represents rates of PPARα mRNA degradation were determined by Monte Carlo simulations (n = 10,000) followed by a conjugate-gradient method implemented in Mathematica.

PPARα oscillates in a circadian manner regulating multiple pathways relating to hepatic lipid metabolism. Similar to the gene expression patterns of the clock components, PPARα did not differ between lean and obese dam offspring fed control diet (**Figure 3A**). However, AUC values for PPARα suggested that offspring of obese dams have an exacerbated response when fed HFD by displaying the lowest AUC values among groups, similar to the mRNA expression of core clock machinery (**Table 1**). These findings were consistent with our previous reports showing decreased activation of PPARα targets in offspring of obese dams [28].

### Mathematical modeling of PPARα time changes

We utilized mathematical modeling of PPARα mRNA expression to identify mechanistic parameters that drive alterations in PPARα rhythm [52], [53]. The model consisted of a differential equation with eight parameters that could contribute to the expression or regulation of PPARα and are defined in **Table S3 in File S1**. The fitted model for PPARα relative mRNA expression is shown in **Figure 3C and 3D**. An optimal value for each parameter was determined by Monte Carlo simulations (n = 10,000) followed by a conjugated gradient method (**Table S4 in File S1**). Partial Rank Correlation Coefficients (PRCC) was performed to determine the predominant contribution of each parameter in the model.

PRCC revealed three parameters were consistently significant for the PPARα model (see **Table S5 in File S1** for the PRCC values), which included *v_p_*, *K_p_*, and *δ_p_* (p<0.001 for all parameters). **Figure 3E and 3F** show the estimated values for *v_p_* and *δ_p_* where *v_p_* represented the maximum rate of mRNA synthesis and *δ_p_* non-specific rates of mRNA degradation. Decreased *ν_p_* values, shown in **Figure 3E**, indicated that the maximum rate of PPARα mRNA synthesis is decreased due to maternal obesity. **Figure 3F** showed a stepwise increase of *δ_p_*, which suggests that obese-HFD offspring have the highest levels of non-specific PPARα mRNA degradation. *K_p_* represented the activation constant for enhancement of gene expression by BMAL1 (**Table S5 in File S1**).

### Histone modifications near the PPARα transcription start site

**Figure 4:**
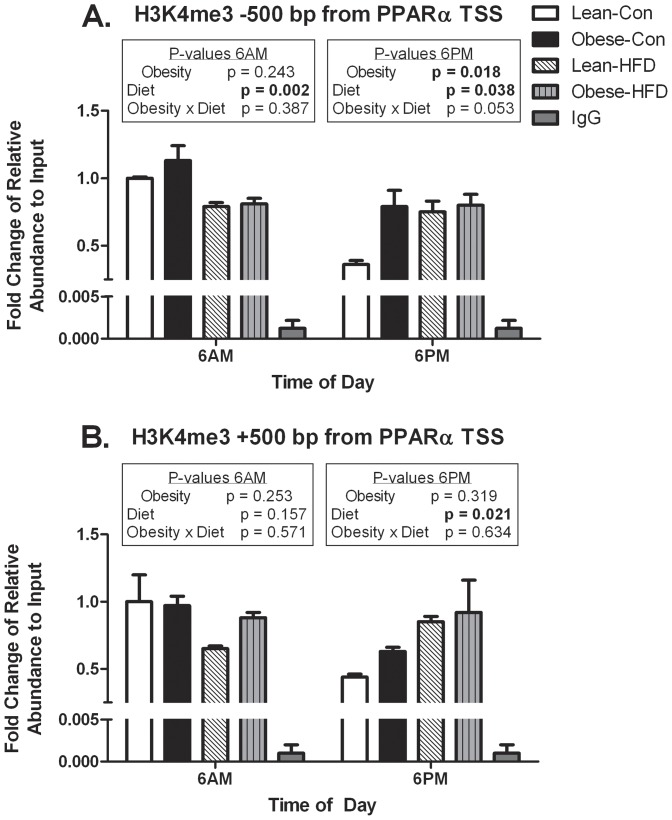
H3K4me3 enrichment on the PPARα promoter. Chromatin immunoprecipitation for H3K4me3 was carried out and A) upstream (−500 base pairs) and B) downstream (+500 base pairs from TSS) regions of the PPAR-α gene were amplified in pools of liver samples (each pool represents 3–5 separate animals) from offspring of lean or obese dams fed either control or HFD (run in triplicate). Enrichment was determined by real time RT-PCR and normalized to input levels. Data are presented as mean ± SEM. Statistical differences were determined using a two-way ANOVA to examine the effects of maternal obesity and post-weaning HFD. Significant interactions were followed by one way ANOVA and Student-Newman-Keuls *post hoc* analyses (P<0.05). Bold values represent significant main effects and interactions and values with different letter superscripts are significantly different from each other (P<0.05).

**Figure 5:**
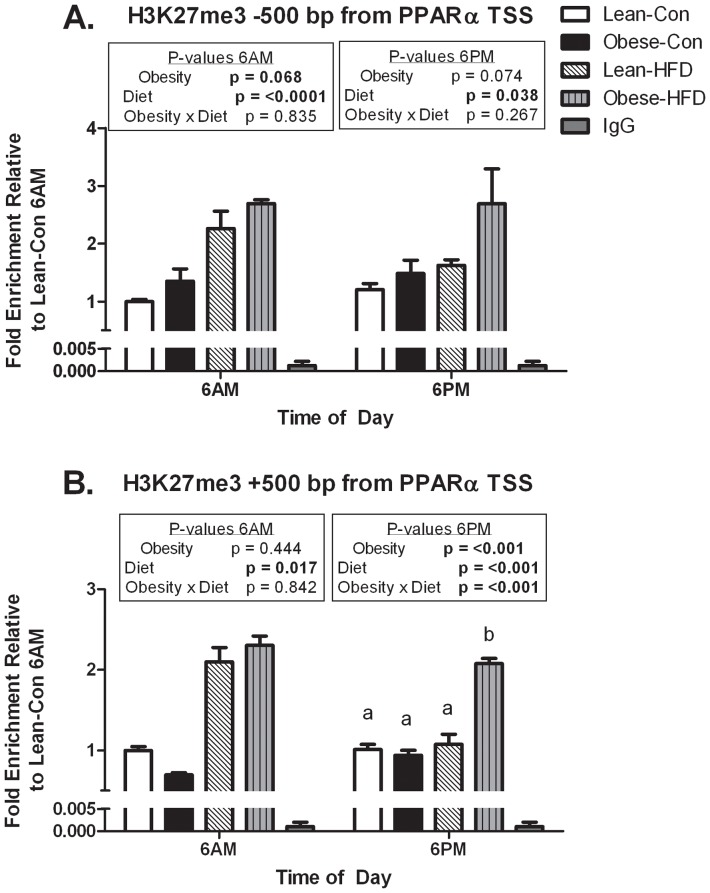
H3K27me3 enrichment on the PPARα promoter. Chromatin immunoprecipitation for H3K27me3 was carried out and A) upstream (−500 base pairs) and B) downstream (+500 base pairs from TSS) regions of the PPAR-α gene were amplified in pools of liver samples (each pool represents 3–5 separate animals) from offspring of lean or obese dams fed either control or HFD (run in triplicate). Enrichment was determined by real time RT-PCR and normalized to input levels. Data are presented as mean ± SEM. Statistical differences were determined using a two-way ANOVA to examine the effects of maternal obesity and post-weaning HFD (P<0.05). Significant interactions were followed by one way ANOVA and Student-Newman-Keuls *post hoc* analyses (P<0.05). Bold values represent significant main effects and interactions and values with different letter superscripts are significantly different from each other (P<0.05).

The TTL, as mentioned previously, involves dynamic transcription factor binding to gene promoters of core clock machinery and metabolism-related targets over the course of the day. Assessing chromatin states offers insight into whether gene transcription was activated or repressed. Here, we focused on PPARα transcription at the start of the light (6AM) and dark (6PM) cycles. Accordingly, ChIP was performed on the PPARα promoter to assess whether the effects of maternal obesity and HFD on PPARα mRNA expression were related to epigenetic changes, specifically histone modifications. ChIP was performed for an active gene transcription mark (H3K4me3) and a silencing transcription mark (H3K27me3) in liver at 6AM and 6PM. Quantitative RT-PCR was performed ±500 base pairs from the PPARα TSS to assess the histone modification status of H3K4me3 and H3K27me3 as these two histone marks are bivalently located near the TSS as shown in **Figure 4** **and** **Figure 5**.

H3K4me3 showed a divergent pattern in control-fed offspring of lean and obese dams, displaying higher enrichment occurring at 6AM and lower at 6PM, both upstream (−500 base pairs) and downstream (+500 base pairs) from the PPARα TSS (**Figure 4**). Lean and obese dam offspring fed HFD lacked differential H3K4me3 enrichment differences between 6AM and 6PM upstream from the TSS (**Figure 4A**). The downstream region from the TSS was similar to the upstream region as lean and obese dam offspring fed the control diet displayed differential enrichment (**Figure 4A and 4B**). These findings would suggest that control-fed offspring of lean and obese dams would demonstrate more rhythmic PPARα mRNA expression while HFD-fed groups would lack oscillatory expression. In fact, PPARα mRNA expression was reflective of these histone changes as control-fed offspring of lean and obese dams showed rhythmic expression that was substantially blunted in HFD-fed offspring of lean and obese dams (**Figure 3A and 3B**).

We also assessed the repressive histone mark, H3K27me3. There was a significant effect due to maternal obesity at 6PM +500 base pairs from the TSS, but this effect was driven by the obese-HFD group as shown in **Figure 5B**. Promoter chromatin enrichment of H3K27me3 was significantly higher in HFD-fed offspring of lean and obese dams at 6AM (**Figure 5A**). At 6PM, the obese-HFD group had higher enrichment levels as compared to all other groups (p<0.001) as shown in **Figure 5B**. In addition, at 6AM, enrichment of H3K27me3 at both ±500 base pairs from the PPARα TSS was increased in lean and obese dam offspring fed HFD (**Figure 5A and 5B**). This would suggest that obese-HFD should display the lowest PPARα mRNA expression as enrichment of H3K27me3 as elevated at both 6AM and 6PM and at both TSS locations which is concordant with the PPARα mRNA expression shown in **Figure 3A and 3B**.

**Figure 6:**
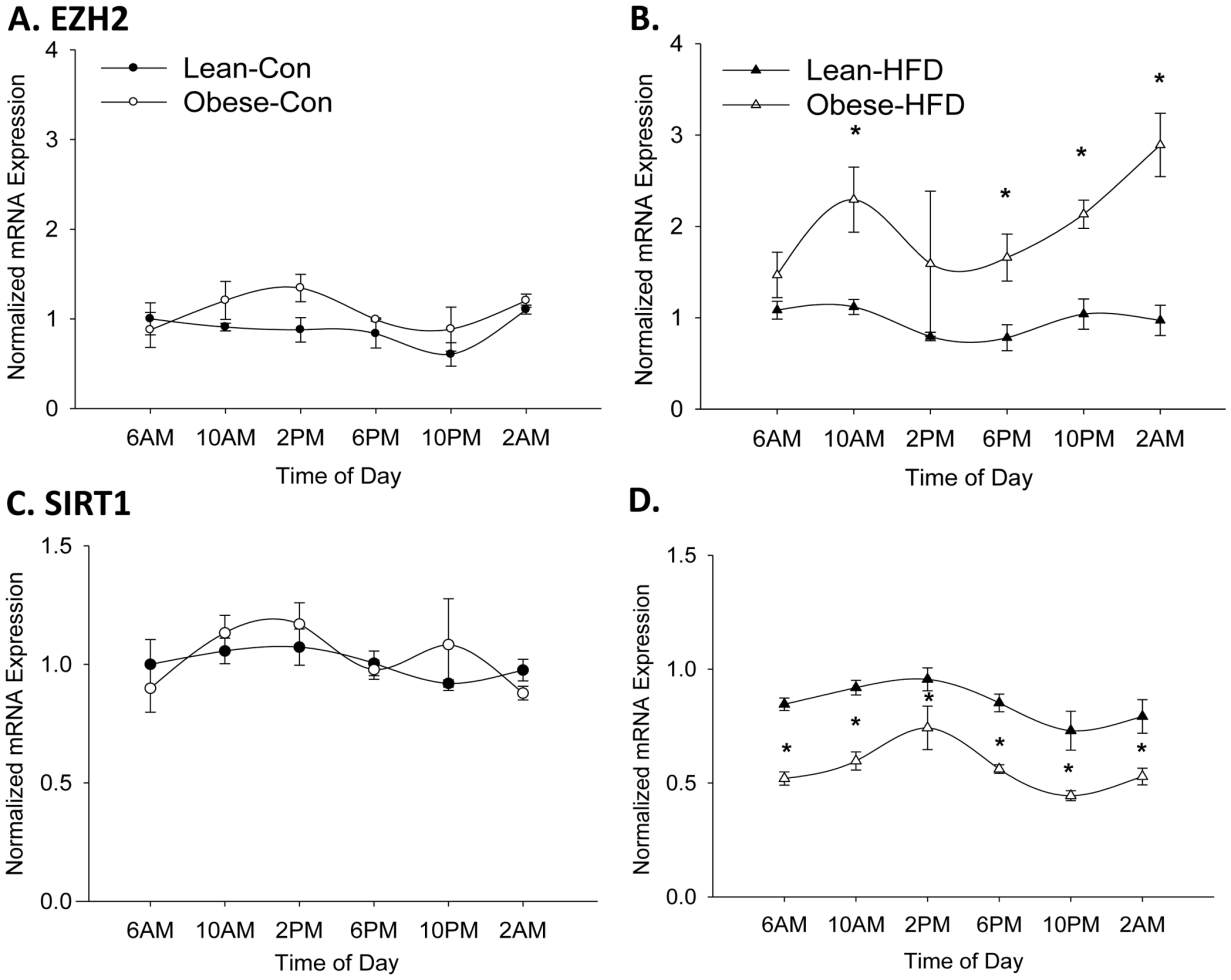
Circadian expression of EZH2 and SIRT1 mRNA. Hepatic mRNA expression of EZH2 in A) Control-fed and B) HFD-fed offspring of lean and obese dams at PND35 (N = 3–5 animals per group, Lean-Con 2AM and Obese-HFD 6AM (N = 5 per group), Lean-Con 10AM and Obese-Con 10PM (N = 3 per group), and all the remaining groups (N = 4)). C) Hepatic mRNA expression of SIRT1 in Control-fed and D) HFD-fed offspring of lean and obese dams at PND35 (N = 3–5 animals per group, Lean-Con 2AM and Obese-HFD 6AM (N = 5 per group), Lean-Con 10AM and Obese-Con 10PM (N = 3 per group), and all the remaining groups (N = 4)). Data are presented as mean ± SEM. Statistical differences were determined using a Student's *t* test. * denotes significance, P<0.05.

Enhancer of zeste homolog (EZH2) is recognized as the primary histone methyltransferase within the polycomb repressive complex (PRC)-2 that mediates H3K27 trimethylation and has also been shown to co-immunoprecipitate with CLOCK and BMAL1 in mouse liver [57]. EZH2 mRNA expression is shown in **Figure 6A and 6B**. There were no differences in levels of expression between lean-con, obese-con, or lean-HFD. However, there was an increase in EZH2 expression at nearly every time point in obese-HFD offspring. AUC values in **Table 1** further support that obese-HFD had the highest EZH2 levels. In addition, EZH2 has been shown to be associated with sirtuin 1 (SIRT1) [58], [59]; SIRT1 deletion leads to increased EZH2 expression levels. Our data coincided with this relationship as SIRT1 levels were reduced (**Figure 6C and 6D**) and EZH2 levels were increased (**Figure 6A and 6B**). Notably, EZH2 mRNA expression was also reflective of H3K27me3 at 6PM (**Figure 5B**) and was consistent with PPARα mRNA expression (**Figure 3A and 3B**).

## Discussion

The maintenance of diurnal circadian rhythm is necessary for normal metabolic function. Misalignment of activities of the molecular clock such as oscillatory disruption in the period, phase, or amplitude of core clock components are associated with the development of metabolic diseases, including obesity, diabetes, and cardiovascular dysfunction [37], [60]. Moreover, alterations in diet composition (high fat diets), timing of eating (night eating), or life-style factors (sleep deprivation or night-shift work) are known to alter circadian rhythms and impair metabolism. However, the influence of gestational experiences (such as maternal obesity) on circadian rhythm in the offspring remains poorly understood. Since, maternal diet and obesity during pregnancy have been shown to influence offspring metabolism, appetite, and adiposity; altered circadian rhythms could be causal mediators.

Although circadian disruptions in periodicity or phase were not apparent in the present report, our findings unequivocally demonstrated that oscillatory amplitude of both core clock regulators and circadian metabolic regulators were altered in offspring when challenged with HFD that was exacerbated in offspring of obese dams. In particular, exposure to both maternal obesity and 2 week post-weaning HFD challenge resulted in the most impairment of gene transcription of core clock machinery and metabolic and epigenetic targets. Changes in PPARα mRNA expression were specifically linked to decreased rates of mRNA synthesis and increased rates of degradation as determined by mathematical modeling. Additionally, epigenomic changes were evident via differences in enrichment of H3K4me3 and H3K27me3 histone marks on the PPARα promoter. In agreement with mRNA expression, the coupling of maternal obesity and HFD exposure led to the most dramatic changes in our mathematical model and histone mark enrichment.

In addition to its central role as a pleiotropic regulator of lipid metabolism, PPARα directly interacts with core clock components in a circadian fashion. Dietary challenges such as fasting and HFD lead to increased PPARα as a normal adaptive response aimed at shunting lipids toward oxidation and away from storage. In an ongoing study, where offspring were fasted for 24 h (at PND22), we observed an attenuated induction in PPARα mRNA and nuclear protein expression in obese dam offspring compared to lean dam counterparts (data not shown). These findings clearly indicate that maternal obesity impairs the underlying mechanisms regulating transcriptional induction of PPARα. Moreover, we have previously demonstrated that exposure to maternal obesity leads to reduced mRNA expression of PPARα target genes prior to the development of obesity or adiposity gains [28]. Consequently, offspring of obese dams are unable to adequately mount a response to metabolic demands that require mobilization of lipids (viz. fasting and high fat diets); specifically, via an inability to induce PPARα and its downstream targets. We have previously shown that another critical regulator of hepatic fatty acid oxidation, SIRT3, was reduced at both the transcript and mitochondrial protein levels in offspring of obese dams [30]. Hence, our studies indicate that complementary pathways involved in hepatic lipid metabolism (PPARα and SIRT3) are impaired by maternal obesity. These may, in concert, contribute to increased offspring susceptibility to nonalcoholic fatty liver disease at weaning [28], [30] and development of obesity in later life [22], [61]. Nevertheless, our previous work did not investigate mechanisms related to changes in PPARα transcription. Indeed, there are limited studies regarding control of PPARα transcription and most research has focused on PPARα-mediated transactivation of downstream targets.

The use of a mathematical model offered valuable insight into the magnitude that distinct biological mechanisms played in modifying PPARα mRNA expression. We identified that mRNA synthesis and degradation rates were mechanisms involved in modulating mRNA expression of PPARα (**Figure 3E and 3F**). Notably, obese-HFD offspring had the highest rates of degradation and the lowest PPARα mRNA expression. These findings suggest an imbalance between transcription and mRNA degradation. There are specific mechanisms associated with the coupling of initiation and decay of mRNA which include mRNA imprinting, alternative transcription start site, alternative splicing, alternative poly-adenylation, and promoter-regulated decay as recently described in a review by Haimovich et al. (2013) [62]. Each mechanism has different factor(s) that regulate mRNA stability and mRNA decay according to location, binding sites, and timing. It is likely that one or more of these specialized mechanisms are influenced by maternal obesity. The mathematical model was limited to the eight parameters that were chosen *a priori*; it is likely that other mechanisms, not included in the model, may have also contributed to changes in PPARα mRNA expression. Nevertheless, this model was chosen due to its capacity to detect parameters contributing to mRNA expression due to alterations in circadian rhythmicity. A limitation of the study, but not the model, was that we did not experimentally evaluate rates of PPARα mRNA synthesis and decay. While identifying the precise machinery involved with mRNA stability and degradation was outside of the scope of the current study, these certainly warrant further investigation.

The prevailing hypothesis in developmental programming is that gestational events alter epigenetic and epigenomic regulation of key genes [63]–[66]. While the initiating signals whereby maternal obesity influences epigenetic regulation of genes remains unclear, increasing information about epigenetically regulated metabolic targets genes is forthcoming. Embryonic development and differentiation are associated with large changes in the epigenomic landscape [67]. In fact, recent evidence suggests that key physiological patterns such as circadian rhythms are associated with highly coordinated alterations in epigenetic histone marks and transcription factors [60], [68]–[70]. Consequently, via ChIP, we assessed if maternal obesity and HFD led to differences in histone demarcation (H3K4me3 and H3K27me3) on the PPARα promoter that were associated with changes in gene expression. H3K4me3 is known to follow a rhythmic pattern of enrichment at TSSs and a lag time between H3K4me3 and permissive gene transcription has been reported [68] and our findings supported this notion (**Figures 4A and 4B**). Although our H3K4me3 findings offer valuable insight into the HFD-induced reduction in PPARα mRNA amplitude, they do not fully explain the exacerbated suppression of mRNA expression in the obese-HFD group. It has been reported that H3K4me3 may be more strongly correlated with increasing gene expression in low CpG promoters as compared to high CpG promoters [71]. PPARα is considered a high CpG promoter which may be a possible explanation for the reason H3K4me3 was not as indicative of HFD-induced changes in PPARα mRNA expression. However, the rhythmic pattern of PPARα mRNA expression can, in part, be explained by H3K4me3 enrichment near the TSS.

Polycomb repressive complexes (PRC)1 and PRC2 mediate the developmentally important repressive mark H3K27me3. PRC1 and PRC2 have crucial roles in pattern specification, organ development, and cellular proliferation and differentiation [72]–[74]. The histone methyltransferase EZH2 is part of PRC2 and the sole enzyme that methylates H3K27me3, but it has also been shown to be involved in the maintenance of circadian clock machinery. Knockdown of EZH2 disrupts circadian rhythm and has been linked to changes in H3K27me3 binding to clock gene promoters [57]. More recently, there is evidence that reduced SIRT1 leads to increased stability and expression of EZH2 [59]. Our data are consistent with this model, where in response to HFD, a decline in SIRT1 was associated with increased expression of EZH2 and thus increased H3K27me3.

Here we showed, H3K27me3 enrichment directly corresponded to the highly suppressive HFD effect on PPARα mRNA expression in both lean-HFD and obese-HFD offspring (**Figure 5A and 5B**). Interestingly, obese-HFD groups showed high levels of enrichment at both 6AM and 6PM as opposed to the lean-HFD rats which only had increased enrichment at 6AM (**Figure 5A and 5B**) suggesting PPARα mRNA expression may be more suppressed by HFD in obese-dam offspring. Indeed, PPARα mRNA expression was lowest in the obese-HFD group (**Figure 3B** **and** **Table 1**). These findings suggest that maternal obesity and HFD are more strongly related to epigenetic changes associated with gene silencing, rather than the repression of gene activation. These results are also in agreement with our modeling data which indicated increased rates of mRNA degradation. In physiological terms, this relationship makes sense as well, as increased EZH2 is associated with adipogenesis and reduced SIRT1 is linked to decreased energy expenditure. Although we did not measure markers of adipogenesis or energy expenditure in this study, we have recently demonstrated increased adipogenesis in white adipose tissue [61] and decreased energy expenditure in obese dam offspring [30]. Our epigenetic changes are in agreement with other reports that found maternal HFD [40], [75] and maternal protein restrictions [76], [77] led to histone modifications.

There are a few limitations to the current study. We did not monitor feeding behavior or cage activity which are both known to be associated with circadian rhythmicity [78]–[81]. We have previously shown that exposure to maternal obesity does not alter food intake in offspring on either a control or HFD [22]; however, it is possible that the timing of food intake or activity is altered which is associated with circadian disruption. In addition, the present studies focused solely on the effects of maternal obesity or HFD on offspring liver and did not examine effects on the central clock. Each of these certainly warrants further investigation in future studies. It is also worthy to note that recent studies have examined the influence of maternal HFD-induced obesity on gene regulation of circadian rhythms. These studies conducted in non-human primates also found a robust effect of maternal and post-natal HFD on expression of the Clock paralog, *Npas2* [40] and SIRT1 expression and acetylation [82]. In contrast to our findings, these effects were mainly driven by maternal HFD and not by maternal obesity *per se*, as revealed by diet-reversal during gestation. While the precise reasons are unclear, differences in diet composition to produce obesity, degree of maternal adiposity, exposure to maternal diets during lactation, and species differences may certainly contribute to differences in findings to those observed in our model.

Collectively, our results indicated that the combination of maternal obesity and HFD led to the greatest disruption of core clock machinery and reductions of PPARα mRNA expression. Mathematical modeling revealed that exposure to maternal obesity led to decreased mRNA synthesis rates and a stepwise increase in mRNA degradation, with obese-HFD displaying the highest rates of mRNA degradation. Epigenomic changes in H3K4me3 appeared to contribute to the rhythmic expression of PPARα in control-fed groups and blunted rhythmic expression in HFD-fed groups while H3K27me3 appeared to play a greater role in HFD-induced effects in the presence or absence of maternal obesity. In conclusion, exposure to maternal obesity *in utero* and post-weaning HFD appear to “poise” offspring to be hyper-responsive to HFD promoting development of obesity in later life.

## Supporting Information

File S1 **SUPPLEMENTARY MATERIALS AND METHODS. FIGURE S1. Serum Parameters at 6AM. TABLE S1. Primers Sequences for Real-time RT-PCR Analyses. TABLE S2. Animal Characteristics of Offspring of Lean and Obese Dam Offspring at 6AM. TABLE S3. Model Parameters and Definitions. TABLE S4. Estimated PPARα Parameter Values. TABLE S5. Most Highly Correlated Model Parameter PRCC Values.**
(DOCX)
