# Supplementary material for: High Fat Diet and In Utero Exposure to Maternal Obesity Disrupts Circadian Rhythm and Leads to Metabolic Programming of Liver in Rat Offspring
Source: PLoS One. 2014 Jan 9;9(1):e84209. doi: 10.1371/journal.pone.0084209 (PMC3886966; doi:10.1371/journal.pone.0084209)
Supplement: File S1 — SUPPLEMENTARY MATERIALS AND METHODS. FIGURE S1. Serum Parameters at 6AM. TABLE S1. Primers Sequences for Real-time RT-PCR Analyses. TABLE S2. Animal Characteristics of Offspring of Lean and Obese Dam Offspring at 6AM. TABLE S3. Model Parameters and Definitions. TABLE S4. Estimated PPARα Parameter Values. TABLE S5. Most Highly Correlated Model Parameter PRCC Values. (DOCX) [file pone.0084209.s001.docx]

**SUPPLEMENTARY MATERIALS AND METHODS**

***Serum Measurements.*** Serum insulin, adiponectin, leptin, glucose, nonesterified fatty acids (NEFA), and triglycerides were measured in offspring of lean and obese dams (N = 4-5) at 6AM and 6PM. Serum insulin, adiponectin, and leptin concentrations were assayed using commercially available ELISA (Millipore, Billerica, MA). Glucose in serum was measured using a Beckman glucose analyzer (Beckman Instruments, Fullerton, CA). NEFA concentrations were measured using NEFA-HR reagents (Wako Diagnostics, Richmond, VA). Triglycerides were assayed using a colorimetric assay (Cayman Chemical, Ann Arbor, MI).

***Mathematical Modeling.*** The relative mRNA expression level of PPARα was analyzed with a differential equation as described in Material and Methods. To incorporate the total BMAL1 protein levels into the model, the function$B(t)$ was digitalized according to Leloup and Goldbeter (2004) considering a circadian oscillation of 23.8 hours [43]. The numerical calculations were expedited using a polynomial approximation of BMAL1 protein level obtained with Mathematica (Wolfram Research Inc, Champaign, IL) by the following function:

$B(t)=0.0061388t^{3}-0.241005t^{2}+2.24792t+26.5711\text{ for }0\leq t\leq24$for every 24 h cycle.

The parameter fitting strategy for a given solution X of the differential equation of relative gene expression of PPARα included:

- Minimization of the quadratic distance from the experimental data to the solution X
- (quasi)-periodicity of the solution i.e. $\left| X\left( 24 \right)- X(0) \right|\leq\varepsilon$ for a fixed small positive value ε.
- (quasi)-smoothness of the solution after 24 h i.e. $\left| D¯X\left( 0 \right)- D⁺X(24) \right|\leq\varepsilon$ for a fixed small value of ε > 0.

Parameter values were obtained by Monte Carlo simulations (n = 10,000) followed by conjugate-gradient method implemented in Mathematica.

**SUPPLEMENTARY TABLE S1: Primers Sequences for Real-time RT-PCR Analyses**

| **Gene Name** | **Forward primer (5'- 3')** | **Reverse primer (5'- 3')** |
| --- | --- | --- |
| **BMAL1** | CAGAAGCAAACTACAAGCCAACA | TCGGTCACATCCTACGACAAAC |
| **CLOCK** | GCAATCAAACCCTGGACTGAAT | GGACAACTTGGCCTTGCATATT |
| **Cry1** | CATCAAGAAGCTGGCCACTGA | GCTGCCCACCATTGAGTTCTAT |
| **Cry2** | TTCCCAAGGCTTTTCAAGGAAT | GCCATCTTCATAATGGCTGCAT |
| **EZH2** | CTTTTGTGCCATTGCTAGGCTAA | CCTCAGTGGGAACAGGTGCTAT |
| **Per1** | GGTATTTGGAAAGCTGCAACATTC | CGCCCTCTGCTTGTCATCAT |
| **Per2** | AGGGCATTACCTCCGAGTATATTG | GGCGACTTGGTTGGAGATGTAC |
| **Per3** | CCAGAGCCTTGCTGTCTAACACT | GGCGTGTGTGTTGTGGTAAAAA |
| **PPARα** | ACGATGCTGTCCTCCTTGATG | CAGAATGGCTTCCTCAGGTTCT |
| **PPARα -500 TSS** | AGCCCCGAGGCACTAAATG | GACGGCAGCATCTGATTGG |
| **PPARα +500 TSS** | TTTCCTCATTGAGGCTGACCT | GCTCTGGAGGGCAGAGACATAG |
| **Rev-erbα** | AGGTGGTAGAATTTGCCAAACAC | CACCATCAGCACCTCAAAGGT |
| **RORα** | TACACACCAGCATCTGGCTTCT | GCCACATCACCTCTCTCTGCTA |
| **SIRT1** | CTGTTTCCTGTGGGATACCTGACT | ATCGAACATGGCTTGAGGATCT |

Gene specific primers were designed using Primer Express^™^ Software (Applied Biosystems, Foster city, CA). Real-time PCR reactions were carried out according to manufacturer’s instructions for fast SYBR green master mix and monitored on an ABI Prism 7500 sequence detection system (Applied Biosystems, Foster city, CA) as described under Material and Methods.

| **SUPPLEMENTARY TABLE S2. Animal Characteristics of Offspring of Lean and Obese Dam Offspring at 6AM** | | | | | | | | | |
| --- | --- | --- | --- | --- | --- | --- | --- | --- | --- |
|  |  |  | |  |  | | *P* Values | | |
|  | Offspring of Lean Dams | | | Offspring of Obese Dams | | | Maternal Obesity x Postweaning Diet | Effect of Maternal Obesity | Effect of Postweaning HFD |
| Parameter | Control | | HFD | Control | | HFD |  |  |  |
| Body Weight (g) | 190 ± 6 | | 230 ± 15 | 179 ± 21 | | 216 ± 20 | 0.655 | 0.294 | 0.080 |
| Liver Weight (g) | 10.4 ± 0.4 | | 13.2 ± 1.0 | 10.1 ± 1.6 | | 10.2 ± 0.8 | 0.209 | 0.116 | 0.184 |
| % Liver Weight | 5.3 ± 0.1 | | 5.7 ± 0.1 | 5.6 ± 0.2 | | 4.8 ± 0.5 | 0.368 | 0.474 | 0.745 |
| Fat Pad Weight (g) | 2.4 ± 0.4 | | 4.9 ± 0.8 | 2.4 ± 0.8 | | 3.9 ± 1.1 | 0.554 | 0.550 | **0.021** |
| Data were obtained from male offspring of lean and obese dams at PND35 (N = 3-5 animals per group, Lean-Con 2AM and Obese-HFD 6AM (N = 5 per group), Lean-Con 10AM and Obese-Con 10PM (N = 3 per group), and all the remaining groups (N = 4)). Data are expressed as mean ± SEM. Dams were fed diets via TEN to induce obesity as described in Materials and Methods. Male offspring were cross-fostered at birth to unsurgerized dams fed AIN-93G diet *ad libitum*. At PND21 offspring were weaned onto *ad libitum* HFD (45% of kcals from fat) for a duration of 2 weeks as a metabolic challenge. Weights of liver and fat pads (retroperitoneal plus gonadal fat depots) were assessed at the time of sacrifice. Percent liver weight was calculated as liver weight in grams divided by total body weight in grams. | | | | | | | | | |

**SUPPLEMENTARY FIGURE S1. Serum Parameters at 6AM**

Data were obtained from offspring of lean or obese dams at PND21 (N = 3-5 animals per group, Lean-Con 2AM and Obese-HFD 6AM (N = 5 per group), Lean-Con 10AM and Obese-Con 10PM (N = 3 per group), and all the remaining groups (N = 4)). Data are expressed as mean ± SEM. Statistical differences were determined using a two-way ANOVA to examine the effects of maternal obesity and post-weaning HFD (*P* < 0.05). Two diet main effects were identified (leptin and triglyceride), different letter superscripts denote this main effect (*P* < 0.05). NEFA had a significant interaction identified by two-way ANOVA which was followed by one way ANOVA and Student-Newman-Keuls post hoc analyses (*P* < 0.05). Values with different letter superscripts represent significant differences (*P* < 0.05).

**SUPPLEMENTARY TABLE S3. Model Parameters and Definitions**

| **Parameter** | **Definition** |
| --- | --- |
| $v_{p}\text{ }(\text{nMh}^{-1})$ | Maximum rate of gene $X$ mRNA synthesis |
| $K_{p}\text{ }(\text{nM})$ | Activation constant for enhancement of gene $X$ expression by nuclear BMAL1 |
| $w_{p}\text{ }(\text{nMh}^{-1})$ | Maximum rate of gene $X$ mRNA degradation |
| $K_{q}\text{ }(\text{nM})$ | Michaelis-Menten constant for degradation of gene $X$ mRNA |
| $\delta_{p}\text{ }(\text{h}^{-1})$ | Non-specific degradation rate constant for gene $X$ mRNA |
| $n_{p}$ | Degree of cooperativity of activation of gene $X$ expression by BMAL1 |
| $m_{p}$ | Degree of cooperativity of repression of gene $X$ expression by BMAL1 |
| $\tau_{p}\text{ }(\text{h})$ | Phase drift from normal the BMAL1 period |

**SUPPLEMENTARY TABLE S4. Estimated PPARα Parameter Values**

| **Parameter** | **Lean-Con** | **Obese-Con** | **Lean-HFD** | **Obese-HFD** |  |
| --- | --- | --- | --- | --- | --- |
| $v_{p}\text{ }(\text{nMh}^{-1})$ | 1.165 | 0.866 | 1.493 | 0.899 |  |
| $K_{p}\text{ }(\text{nM})$ | 17.570 | 29.855 | 38.555 | 35.844 |  |
| $w_{p}\text{ }(\text{nMh}^{-1})$ | 1.084 | 0.667 | 0.646 | 1.487 |  |
| $K_{q}\text{ }(\text{nM})$ | 15.599 | 15.468 | 11.906 | 8.467 |  |
| $\delta_{p}\text{ }(\text{h}^{-1})$ | 0.058 | 0.184 | 0.355 | 0.618 |  |
| $n_{p}$ | 3.318 | 11.640 | 5.004 | 5.061 |  |
| $m_{p}$ | 5.924 | 9.848 | 3.687 | 10.815 |  |
| $\tau_{p}\text{ }(\text{h})$ | 20.745 | 17.576 | 15.370 | 14.010 |  |

**SUPPLEMENTARY TABLE S5. Most Highly Correlated Model Parameter PRCC Values**

| Parameter | Lean-Con | | Obese-Con | | Lean-HFD | | Obese-HFD | |  |  |
| --- | --- | --- | --- | --- | --- | --- | --- | --- | --- | --- |
|  | 6 AM | 6 PM | 6 AM | 6 PM | 6 AM | 6 PM | 6 AM | 6 PM |  |  |
| $v_{p}$ | 0.91 | 0.96 | 0.57 | 0.72 | 0.58 | 0.75 | 0.48 | 0.75 |  |  |
| $K_{p}$ | -0.77 | -0.83 | -0.95 | -0.95 | -0.95 | -0.97 | -0.97 | -0.97 |  |  |
| $\delta_{p}$ | -0.76 | -0.75 | -0.65 | -0.68 | -0.68 | -0.72 | -0.64 | -0.67 |  |  |
